# A survey of mental health professionals in a paediatric hospital during COVID-19

**DOI:** 10.1177/13591045211033186

**Published:** 2022-01

**Authors:** Brian CF Ching, Sophie D Bennett, Isobel Heyman, Holan Liang, Matteo Catanzano, Kate Fifield, Zoe Berger, Suzanne Gray, Emma Hewson, Mandy Bryon, Anna E Coughtrey, Roz Shafran

**Affiliations:** 1 UCL Great Ormond Street Institute of Child Health, 11700 University College London, London, UK; 2 Psychological and Mental Health Services, 4956 Great Ormond Street Hospital for Children NHS Foundation Trust, London, UK

**Keywords:** Adolescence, chronic illness, mental health professionals, paediatrics, questionnaires

## Abstract

**Background:**

There is little understanding of the mental health impact for young people with long-term physical health conditions and mental health professionals’ experiences of supporting them during COVID-19. This service evaluation aimed to conduct a survey of the psychological services provided by mental health professionals in a paediatric hospital in relation to COVID-19.

**Method:**

Clinical psychologists and assistant psychologists (*n* = 76) across the hospital were asked to complete a survey, asking about their perceptions of COVID-19’s impact on patients and families and experiences of providing support during COVID-19. Open-ended survey questions were analysed qualitatively using framework analysis.

**Results:**

Respondents described perceived impacts on patients and families around social isolation, school closure, family relationships, physical health, mental health, treatments and social support. Respondents’ experiences of providing mental health support during COVID-19 highlighted themes around providing remote/virtual support, workload and facilitators and barriers to their work.

**Conclusions:**

Mental health professionals surveyed reported a complex mental health landscape in young people with long-term physical health conditions and their families during COVID-19. Service-wide involvement is needed to facilitate changes to support vital adaptations to remote/virtual working. Research on the mental health of young people with long-term physical health conditions and staff experiences of providing support is warranted.

## Introduction

Young people and their families have seen many significant life changes since the global outbreak of the coronavirus disease (COVID-19), from school closures and social distancing measures, to an economic recession and health crisis. As a result, the mental health of young people and their families has been hugely affected. A nationally representative survey in the United Kingdom found that since the pandemic, one in six young people aged 5 to 16 had a probable mental health disorder compared to one in nine in 2017 (NHS Digital, 2020).

Factors like social isolation, family confinement and pre-existing mental health difficulties may be risk factors for poor mental health and mental health disorders in young people during the pandemic (Guessoum et al., 2020). However, there is a scarcity of research on the mental health impact for young people with long-term physical conditions (LTCs) despite them being a vulnerable group to both COVID-19 (Glasper, 2020; Jones et al., 2020) and mental health difficulties (Glazebrook et al., 2003; Verhoof et al., 2014), especially during COVID-19 (Aishworiya & Kang, 2020; Patel, 2020).

Examinations of the impact of COVID-19 on children’s services have also been scant. Although we have seen wide-spread changes in child health services (Badawy & Radovic, 2020; Barney et al., 2020; Chanchlani et al., 2020), there is very little research on mental health professionals’ experiences of providing support for young people with LTCs and their families’ mental health during COVID-19. As evidence suggests hospital staff may experience mental health difficulties as a result of working during the pandemic (Greene et al., 2021; Lai et al., 2020), it is important to consider staff experiences during this time. Such data will be important to inform adaptive changes in service provision to address mental health needs that arise as a result of COVID-19 itself or the impact of measures taken to manage the virus.

The aim of this service evaluation was to conduct a survey of the provision given by the psychological services in a paediatric hospital in relation to COVID-19. Specifically, the survey aimed to collect data on clinical psychologists (CPs) and assistant psychologists’ (APs) perspectives of the impact of COVID-19 on patients and families and experiences of providing mental health support during the pandemic to inform patient needs and service improvements.

## Methods

### Approval

The Great Ormond Street Hospital for Children NHS Foundation Trusts’ Clinical Audit Team approved this survey as part of a service evaluation (audit reference number: 2873). Although it was decided that the collection and analysis of data did not require ethical review by an NHS research ethics committee, care was taken to ensure ethical standards were met. Respondents were assured that their response to the survey was confidential and anonymised. No personally identifiable information was collected or described in this study.

### Sample

All 76 non-medical psychology staff in the hospital’s psychological services were invited to participate (63 CPs and 13 APs). In the United Kingdom, APs will have at least completed an accredited 3-year undergraduate degree in psychology and experience in working in clinical and/or research settings. CPs have at least also completed an accredited 3-year postgraduate doctorate in clinical psychology and be registered with the Health and Care Professions Council.

### Setting

The hospital is a specialist paediatric hospital in London, United Kingdom, that provides outpatient and inpatient services for young people with a variety of physical health needs, which are usually complex and long term. There is a large psychological and mental health service within the hospital providing embedded and accessible mental health support to patients and their families across all physical health specialities. In response to the outbreak, the hospital has implemented specific measures and policies to protect patients, families and staff, and to control the spread of the virus on hospital grounds.

### Procedure

Responses from all CPs and APs in the hospital were sought through purposive sampling. All CPs and APs in the hospital email address book were sent a personalised email with the link to the survey. This was followed by a follow-up email one week later. As responses were anonymous, all CPs and APs received the follow-up email even if they had completed the survey. Those who were on leave were contacted on their return.

### Surveys

Two questionnaires (one for CPs and one for APs) were developed by the research team and clinical team leads through iterative discussion during the design of the service evaluation. The clinical team’s experiences of working during the pandemic and their understanding of COVID-19’s impact on young people and their families, combined with the research team’s background of questionnaire development, were combined to develop the questions used in the service evaluation. The final versions were hosted on the Qualtrics platform (see Supplemental Appendices 1 and 2).

Structured and open-ended questions were used to gather rich insight into respondents’ diverse perspectives and experiences of providing psychological support to patients and families during COVID-19. A branching structure was used where respondents were first asked to provide single responses with ordinal and nominal categories on the proportion of patients being seen for COVID-specific mental health needs and its impacts on patients (school, family life, friendships, treatment of physical illness and their own and their parents’ mental health). A scaled question was used to assess the average proportion of session time that was used to address COVID-specific concerns or impact on a scale from 0 to 100. This was followed by respondents being asked to provide multiple responses with nominal categories on the types of support that was provided for COVID-related difficulties, including low intensity and high intensity psychological interventions. Open-ended questions were used to elicit more in-depth data on respondents’ perception of the main impact of COVID-19 on patients and their experiences of supporting these patients and families. It also provided opportunity for respondents to include responses that were not included as options in the structured questions.

Respondents were asked to give estimated answers to questions to reduce time burden. Respondents could skip questions, and none were compulsory. The major difference between the two questionnaires was that APs were only asked about low intensity treatment when asked about the support that was provided for COVID-related difficulties as APs do not have the training to deliver high intensity interventions. In this context, low intensity treatments comprise of six hours or less treatment time (using self-help materials, such as low intensity cognitive behavioural therapy (CBT)) delivered by a trained practitioner or supporters, such as APs, and high intensity treatments include full treatment of CBT delivered by individuals with professional qualifications, such as CPs (Shafran et al., 2021). Additional questions about their low intensity cases were included, such as where referrals came from and whether standardised measures were used. Depending on eligibility for branching questions and respondents’ role, respondents were asked between 13 and 21 questions. The questionnaires took approximately 5–10 minutes to complete. Anonymity and confidentiality were ensured by not asking respondents any personal or identifying information.

### Analysis

Qualitative data were analysed using framework analysis (Gale et al., 2013). Two researchers (BCFC and KF) familiarised themselves with the data through immersion, by reading the qualitative responses line by line. Notes were made on initial codes that seemed important or relevant. Initial responses were used to form a preliminary analytical coding framework. Codes were compared and the working analytical framework was developed together. The framework was then applied to the data by BCFC where responses were indexed using the working codes and categories. Throughout the process, codes and categories would be added and changed as new ideas were identified from the data. Memos about deeper meanings of the data were also kept as they were identified. After the framework was applied, the matrix was developed in tables to summarise the distinct categories, presented in the Results. The data were interpreted to descriptively elucidate meaning and identify patterns and differences. This was reviewed by members of the research team.

## Results

Data were collected between August 14, 2020 and October 12, 2020. A total of 48 (63.2%) people completed at least one question and 45 (59.2%) completed it fully. We report findings for the 48 respondents. The response rates across the two groups did not differ greatly; we received responses from 39 (61.9%) clinical psychologists and 9 (69.2%) assistant psychologists. One hundred and five responses to open-ended items were included. This yielded 2,937 words for qualitative analysis.

### Impact of COVID-19 on patients and families

Respondents provided an estimated proportion of referrals that were for mental health difficulties specifically related to the current pandemic. Most respondents rated ‘none’ (62.5%), followed by ‘some’ (29.2%) and ‘about half’ (8.3%). No respondents rated the proportion of COVID-related referrals as being ‘most’ or ‘all’ of the referrals.

Respondents’ ratings of the perceived impact of COVID-19 on patients and families’ lives were diverse, summarised in Table 1. Across the domains, patients’ friendships, treatment of patients’ physical illnesses and parents’ mental health were most rated to be negatively impacted by COVID-19. Family life, patients’ mental health and school were most perceived to be impacted both negatively and positively. A small proportion of respondents perceived the domains to be more positively impacted by the pandemic, except for parents’ mental health which is counter to expectation.

**Table 1.** Respondents’ perceived impact of COVID-19 on patients and families.

	*N*	*n* rated more negative (%)	*n* rated both negative and positive (%)	*n* rated more positive (%)	*n* rated do not know (%)
School	46	21 (45.7)	21 (45.7)	4 (8.7)	0 (.0)
Family life	46	9 (19.6)	34 (73.9)	3 (6.5)	0 (.0)
Friendships	44	32 (72.7)	10 (22.7)	2 (4.5)	0 (.0)
Treatment of physical illness	37	21 (56.8)	13 (35.1)	2 (5.4)	1 (2.7)
Patients’ mental health	46	11 (23.9)	31 (67.4)	3 (6.5)	1 (2.2)
Parents’ mental health	45	25 (55.6)	20 (44.4)	0 (.0)	0 (.0)

Qualitative analyses were conducted on responses from two open-ended questions where themes and sub-themes emerged from the analyses, summarised in Tables 2 and 3.

**Table 2.** Themes and sub-themes of respondents’ perceived main impact of COVID-19 on patients and families.

Theme	Sub-theme
Social isolation	Disconnect from the outside world
	Loss of routine
School closure	Access to education
	Reduced school-specific stressors
	Exams
	Anticipatory anxiety of returning to school
Family relationships	Pressure on the household
	Improved relationships
Physical health	Improvements in physical health
	Worsening of physical health
	COVID-19 hospital admissions
Mental health	Anxiety
	One carer policy
	Increased parental burdens
	Feelings of being different
	Low mood
	Behavioural difficulties
	Improvements in mental health
Treatments	Reduced quality
	Medical treatments
	Psychological treatments
Social support	Reduced access to established social support systems
	Reduced access to social/community services
	Cultural stigma

**Table 3.** Themes and sub-themes of respondents’ experiences of providing support to patients and families during COVID-19.

Theme	Sub-theme
Remote/virtual support	Expectations
	Engagement
	Access to psychological support
	Technological challenges
	Distrust in remote/virtual working
Workload	Increasing workload
	Adapting to patient needs around COVID-19
Facilitators and barriers	Teamwork and communication
	Motivation
	Electronic system
	Lack of face-to-face care
	Reduced input from other services

An open-ended question asked about respondents’ perceived main impact of COVID-19 on patients and families. Responses indicated a range of perceived impacts (see Supplemental Appendix 3 for further details and quotes):

#### Social isolation

Many patients and families were perceived to be socially isolated, which impacted how connected they felt with the outside world, including with their friends and families. This was perceived to result in a loss of routine as patients and families were unable to continue their usual way of life.

#### School closure

Many patients and families were perceived to be impacted by school closure. Respondents reported many families struggling with accessing education through homeschooling and virtual schoolwork without the usual learning support in place, while others reported having positive experiences. Some patients’ well-being was perceived to have improved, however, due to reduced school-specific stressors, such as bullying and performance anxiety. Not needing to sit exams this year was reported to have had a mixed effect as respondents described some patients feeling worried about exam results while others were relieved. Some respondents also described families being worried about the transition back to school.

#### Family relationships

Family relationships were mostly perceived to be strained due to more time spent together with little opportunity for private space. However, a minority of respondents said some families’ relationships improved due to spending more time together.

#### Physical health

Patients’ physical health was perceived to have been both positively and negatively impacted. Some families were perceived to have had more time to manage their physical health, which improved patients’ conditions. On the contrary, a few respondents described worsening of existing conditions because of reduced activity levels. Patients who contracted the virus were also reported to need to manage the health implications.

#### Mental health

With regards to patients’ and parents’ mental health, the largest difficulty that was perceived by respondents was anxiety. Anxiety revolved around catching the virus, spreading the virus to vulnerable family members, their current physical health and trauma of being hospitalised during the pandemic. Families were perceived to experience distress related to the one carer policy in the hospital, which initially restricted visits to inpatients to only one carer and no other visitors, including siblings. Some parents were described by respondents to feel overwhelmed during this time. Other mental health difficulties in patients that respondents reported include feeling different from other young people due to the need to shield, low mood and behavioural difficulties. However, some respondents also mentioned perceived improvements in patients’ mental health due to staying at home.

#### Treatments

Respondents perceived a negative impact on interventions for physical and mental health due to the pandemic. Generally, respondents reported a perceived reduction of access to treatments due to hospital restrictions that impacted face-to-face appointments. Delayed access to medical services was perceived to cause a lot of uncertainty for families. Psychological services were thought to also be affected by respondents, notably due to delayed assessments and limitations with remote/virtual psychological work. Access to local child and adolescent mental health services (CAMHS) was reported to be difficult.

#### Social support

Respondents reported that social support for families was negatively affected, both within families and wider established structures of support, such as government policies and third sector services.

### Support for COVID-related difficulties

The median estimated proportion of session time spent on COVID-related difficulties or its impact was 24%. The mode was 20% and responses ranged from 7% to 100% of session time.

CPs (*n* = 39) provided a wide range of support to patients and families for COVID-related difficulties. Respondents were able to select multiple forms of support they provided: one-off supportive telephone calls (33%), low intensity interventions (i.e. fewer than six telephone calls; 15%), signposting (13%) and high intensity interventions (i.e. full course of therapy; 10%). Almost a quarter of respondents reported that no support was needed (22%) specifically for COVID-related difficulties and others reported that other forms of support (7%) were provided, but no detail was elaborated.

Respondents who selected multiple forms of support (*n* = 24) were asked to select the most commonly provided. One-off supportive telephone calls (46%) were reported to be most commonly provided, followed by low intensity interventions (25%), signposting (12%) and high intensity interventions (4%), Similarly, some reported that other forms of support (13%) were most commonly provided without further explanation when asked.

An open-ended question asked about respondents’ experiences of providing support for patients and families during COVID-19. Responses contained common and varied patterns (see Supplemental Appendix 4 for further details and quotes).

#### Remote/virtual support

Respondents’ experiences of remote/virtual sessions were diverse. Some respondents found delivering support via telephone or video sessions a positive experience, whereas others found it came with technological challenges. Respondents reported some families feeling sceptical in the quality of support provided through virtual means as compared to usual face-to-face support. Engagement in patients during remote/virtual appointments was also varied, from having no impact on engagement compared to face-to-face sessions to being challenging for respondents. Likewise, access to psychological support was mixed. Some described the remote/virtual sessions improved access for patients and families due to reasons like not needing to commute to the hospital and feeling safer at home. Contrastingly, others perceived this format reduced access for patients with sensory impairments.

#### Workload

Many respondents described an increased workload from new COVID-related referrals and new presenting difficulties from current patients. Changes in workload were suggested to match changes in governmental policies and regulations; for example, increase in mental health difficulties in patients and families as lockdown restrictions were being lifted. In addition to increased workload, respondents also described a change in their work as a result of patients and families experiencing COVID-related difficulties and having to manage their implications, such as managing uncertainty.

#### Facilitators and barriers

The most prominent facilitators for providing support to families were teamwork and communication, which was described by respondents as important in supporting each other and families within teams in the hospital and external services. Motivation from respondents and electronic systems in the hospital also appeared to be helpful for the support they provided. Lack of face-to-face care was described as a barrier to understanding patients’ difficulties and providing subsequent intervention. Reduced input from external services, like social care, seemed to also limit liaison work respondents could do for patients and families.

#### Low intensity interventions

APs were asked an additional question about the low intensity interventions they provided. Only one had delivered low intensity interventions to support patients and families for COVID-related difficulties. They reported delivering the following low intensity interventions: one-off supportive telephone calls, brief intervention (less than six telephone calls), and signposting.

The other seven APs did not provide low intensity interventions. Two APs reported that some support was needed with providing low intensity interventions to patients and families specifically for COVID-related difficulties. The most frequent reason was that it was beyond their job remit. This was seen in respondents across different teams. Other reasons included patients’ needs being addressed as part of broader consultations, COVID-related difficulties not being the main presenting problem, COVID-related referrals being picked up by other team members and not receiving any COVID-related referrals.

## Discussion

Using both quantitative and qualitative analyses, our findings provided a detailed snapshot of the impact of COVID-19 on the mental health of young people with LTCs and their families that is rooted in staff experiences and direct clinical contact with this population. Most notable is the diversity of perceptions and experiences which illustrate the heterogeneity and complexity of COVID-19’s impact on young people with LTCs’ mental health and how CPs and APs across different teams in a paediatric hospital responded to those varying needs.

### Impact of COVID-19 on patients and families

Our findings show that most CPs and APs did not receive any referrals specifically for COVID-related mental health difficulties, yet discussions around COVID-19 took up about a quarter of session time on average. However, COVID-19 has affected the global population and was the broader context for all patient contacts. The findings may reflect the difficulty of disentangling discussions about COVID-19’s pervasive impact on mental health, physical health and daily life. Such discussions would have reduced the amount of clinical time left for other matters.

Closure of schools was perceived to introduce a range of issues for some young people and families. Access to education and learning was impaired for many, especially for families who have now lost learning support that was provided at schools and now managing remote/virtual schoolwork or homeschooling. Our findings should be viewed in the context of other surveys from different settings. In a survey of nearly 150 children and young people, Barnado’s found 57% of children and young people experienced a decline in school progress during the pandemic (Barnado’s, 2020). In another survey of 3,570 children and young people, those who had a probable mental health disorder were less likely to access support from school or other educational institutions than those who were unlikely to have a mental health disorder (NHS Digital, 2020). It is essential to better understand the positive and negative impacts of COVID-19 in relation to school and more broadly to establish whether COVID-19 has reduced some external pressures and/or whether individuals were able to find some resilience.

The one carer policy was put in place to protect patients and families in the hospital. It was also perceived by professionals to inadvertently be a source of stress for families. As only one parent could visit patients and attend to their care, carers were described as feeling guilty, scared, distressed and overwhelmed by the increased responsibility. Patients’ usual coping strategies and support like siblings and parents were inaccessible during their inpatient stay. This is consistent with previous findings where parents and children who were quarantined or isolated exhibited higher post-traumatic stress symptoms than those who were not (Sprang & Silman, 2013).

### Experiences of supporting difficulties during COVID-19

Challenges with engagement (including technical difficulties) were prevalent in respondents’ experiences of remote/virtual psychological work. Although not the case for all, where some had positive experiences, the issues around remote/virtual psychological work needs to be further explored. A recent survey of mental health professionals found similar results around remote/virtual psychological work posing challenges for staff around building rapport, technical difficulties and reduced capacity for specific psychological treatments (Feijt et al., 2020). On the other hand, a review on interactional differences between telephone and face-to-face psychological therapy suggests there are minimal empirical differences in patient engagement and participation (Irvine et al., 2020). Despite previous research suggesting no significant differences in how patients may experience remotely delivered psychological treatments compared to face-to-face treatments (Irvine et al., 2020), mental health professionals may perceive lower self-efficacy in remote/virtual formats compared to face-to-face work which should be explored.

During this time, respondents saw changes in their work. They saw increased workloads due to both new referrals and current patients experiencing mental health difficulties as a result of COVID-19. The ebb and flow of mental health difficulties were described to mirror changes in governmental policies and regulations. This may reflect preliminary findings showing a steady increase in emotional difficulties in young people in the United Kingdom during the first few months of lockdown (Skripkauskaite et al., 2020). CPs also had to adapt to difficulties that were brought up by the pandemic in patients, such as anxiety about COVID-19 and its uncertainty.

CPs identified several helpful factors in delivering support during COVID-19. Peer support, including teamwork and communication, aided the support they provided for families and professionals. The need for transparent communication was prevalent within immediate teams, but also across other teams in the hospital and with external services. As many services have been overwhelmed during the pandemic as anticipated, reduced input from external services affected the work respondents could do with patients and families, especially around liaison work. Respondents also identified the hospital’s electronic systems to be helpful in organising appointments with patients and families.

### Limitations

Our survey only captures the perspectives of CPs and APs. Insight from other stakeholders, such as psychiatrists, mental health nurses, family therapists, occupational therapists and importantly patients and families themselves, will deepen our understanding of the specific impacts of COVID-19 on young people with LTCs. It is possible that findings were related to specific conditions: some conditions may be more likely than others to be linked with children finding school closure a positive thing, or finding it easier to manage their health condition for example. Future involvement of patients and families should include young people with diverse physical health needs to explore COVID-19’s specific impacts.

Despite employing open-ended questions, respondents were also asked to not spend too much time on completing the questionnaire as the purpose of the survey was to help formulate an initial understanding of CPs and APs’ perceptions and experiences. Data from quantitative and epidemiological studies are needed to systematically assess the mental health of young people with LTCs during COVID-19 and the impact on mental health services. Further qualitative studies are also needed for in-depth exploration of mental health professionals’ experiences of supporting this population during the pandemic.

In addition to the brevity of the qualitative data, they may not fully reflect the quantitative findings. Respondents’ ratings on the survey indicate a nuanced picture where many domains in patients and families’ lives were impacted both positively and negatively, which was not necessarily represented in the quantitative findings to the same degree. Friendships in patients were also not a theme that emerged from qualitative analysis, although reported to be affected by COVID-19 in the quantitative data.

This was a site-specific service evaluation and Great Ormond Street Hospital for Children has a large and long-established specialised clinical psychology service to the paediatric specialities. Our findings may not be generalisable to other paediatric hospitals. Respondents may have unique experiences due to a plethora of factors, such as differences in the regional impact of COVID-19 and resource distribution in hospitals around the United Kingdom. Moreover, the data do not explain the significant heterogeneity of the perceived impact of COVID-19 on patients and families, as well as the experiences of providing mental health support during COVID-19. Our service evaluation cannot highlight the causal mechanisms underlying the diverse experiences of CPs and APs. This needs to be explored in future studies.

### Implications

Initial data on the varying mental health needs of young people with LTCs and families during COVID-19 in a paediatric hospital are highlighted in our survey. Although only capturing one paediatric hospital in the United Kingdom, our findings should be considered along with other data to help understand the mental health ramifications on this vulnerable group.

Providing support for patients and families during COVID-19 has affected respondents in different ways, but many described an increased workload. As the literature has demonstrated worse mental health outcomes in healthcare professionals during COVID-19 (Greene et al., 2021; Lai et al., 2020), the well-being and mental health of CPs and APs should also be considered. More work should complement existing hospital-based services and efforts to support these staff.

It is important to ensure mental health professionals feel confident in this relatively unchartered format of psychological work. Service-wide involvement is needed to facilitate changes to support vital adaptations to remote/virtual working. This may range from specialised training (Austen & McGrath, 2006; McCord et al., 2015) to providing appropriate technology and implementation (Sharma et al., 2020).

Although not a universal experience across teams, our data suggest that most respondents have been delivering briefer forms of support within their routine service. Services need to evaluate how feasible and cost-effective support can be optimised. This may involve APs delivering evidence-based low intensity interventions to ensure CPs have the capacity to address more severe needs. This has been shown to be feasible in a paediatric setting during COVID-19 (Batchelor et al., 2020) with good patient outcomes (Catanzano et al., 2021).

## Conclusions

The findings from this survey illustrate a complex mental health landscape in young people with LTCs and their families during COVID-19 informed by CPs and APs from a paediatric hospital in the United Kingdom. The pandemic was reported to have negative and positive impacts on different domains across patients and families. There is a need for further detailed exploration of this population’s experiences during the pandemic and the impact on their mental health. The diverse experiences of supporting the mental health of young people with LTCs and their families in the pandemic were also highlighted. Research should focus on understanding how these mental health professionals can be supported and include views from other stakeholders.

## Supplemental Material

sj-docx-1-ccp-10.1177_13591045211033186 – Supplemental Material for A survey of mental health professionals in a paediatric hospital during COVID-19 Click here for additional data file. Supplemental Material, sj-docx-1-ccp-10.1177_13591045211033186 for A survey of mental health professionals in a paediatric hospital during COVID-19 by Brian CF Ching, Sophie D Bennett, Isobel Heyman, Holan Liang, Matteo Catanzano, Kate Fifield, Zoe Berger, Suzanne Gray, Emma Hewson, Mandy Bryon, Anna E Coughtrey and Roz Shafran in Clinical Child Psychology and Psychiatry
