# Supplementary material for: A survey of mental health professionals in a paediatric hospital during COVID-19
Source: Clin Child Psychol Psychiatry. 2022 Jan;27(1):122–35. doi: 10.1177/13591045211033186 (PMC8811327; doi:10.1177/13591045211033186)
Supplement: sj-docx-1-ccp-10.1177_13591045211033186 – Supplemental Material for A survey of mental health professionals in a paediatric hospital during COVID-19 [file sj-docx-1-ccp-10.1177_13591045211033186.docx]

**Supporting information – A survey of mental health professionals in a paediatric hospital during COVID-19 – by Ching et al.**

**Appendix S1.** Survey for clinical psychologists.

**Appendix S2.** Survey for assistant psychologists.

**Appendix S3.** Table summarising clinical psychologists and assistant psychologists’ perspectives on the main impact of COVID-19 on patients and families. Quotes for each theme are presented.

**Appendix S4.** Table summarising clinical psychologists and assistant psychologists’ experiences of providing support to patients and families during COVID-19. Quotes for each theme are presented.

**Appendix S1.**

*Survey for clinical psychologists*

Thank you for taking the time to tell us about the support you have been providing to patients and families in response to the mental health needs that have arisen due to COVID-19.

The pandemic has affected all of us in many ways. This survey is about any **SPECIFIC**psychological work you have been doing in relation to COVID-19.

This service evaluation will help capture the COVID-19 related work being undertaken by Psychological Services. We are only interested in estimates so there is no need to go through your caseload in detail.

This survey will take less than 5 minutes to complete. Your responses will be completely anonymous.

1. In all the patients you have seen, roughly what proportion have been referred **SPECIFICALLY** for COVID-related difficulties (e.g. worried about catching the virus or about postponed exams)?
   1. None
   2. Some
   3. About half
   4. Most all
2. Overall, how do you think COVID-19 has impacted your patients in the following domains?
   1. School
      1. More negative
      2. Both negative and positive
      3. More positive
      4. Don’t know
   2. Family life
      1. More negative
      2. Both negative and positive
      3. More positive
      4. Don’t know
   3. Friendship
      1. More negative
      2. Both negative and positive
      3. More positive
      4. Don’t know
   4. Treatment of Physical illness
      1. More negative
      2. Both negative and positive
      3. More positive
      4. Don’t know
   5. Their own mental health
      1. More negative
      2. Both negative and positive
      3. More positive
      4. Don’t know
   6. Their parent’s mental health
      1. More negative
      2. Both negative and positive
      3. More positive
      4. Don’t know
3. Overall, what do you think was the main impact of COVID-19 on your patients?

E.g., not being able to see your face due to PPE, worrying about catching the virus, school closure, postponed school exams, delayed surgery, delayed psychological assessment/treatment, no longer being able to have visitors in the hospital *(open question)*

1. Although we are aware it varies for different patients, for those patients with **SPECIFIC**concerns related to COVID-19 or its impacts (e.g., catching the virus, missing exams), on average what proportion of session time is spent on those concerns? (*Slider 0-100)*

0 would indicate none of the session time

50 would indicate half of the session time

100 would indicate all of the session time

1. What kind of support are you providing **SPECIFICALLY** for COVID-related difficulties? Please select all that apply.
   1. One-off supportive telephone calls
   2. A few (less than 6) telephones call of 20-30 minutes or less
   3. Full course of therapy (more than 6 sessions; typically, one hour)
   4. Signposting
   5. Other
   6. Not needed
2. If you selected multiple types of support, which was the most common support provided?
   1. One-off supportive telephone calls
   2. A few (less than 6) telephones call of 20-30 minutes or less
   3. Full course of therapy (more than 6 sessions; typically, one hour)
   4. Signposting
   5. Other
   6. Not applicable
3. If you selected 'Other', please explain what you mean in the box below. *(open question)*
4. Is there anything else you would like to tell us about the support you have been providing to patients and their families during COVID-19? *(open question)*
5. Lastly, what medical stream are you from? *(open question)*

Thank you for your input!

If you have any questions, please feel free to drop us a message at MentalHealth.Access@gosh.nhs.uk

**Appendix S2.**

*Survey for assistant psychologists*

Thank you for taking the time to tell us about the support you have been providing to patients and families in response to the mental health needs that have arisen due to COVID-19.

The pandemic has affected all of us in many ways. This survey is about any **SPECIFIC**psychological work you have been doing in relation to COVID-19.

This service evaluation will help capture the COVID-19 related work being undertaken by Psychological Services. We are only interested in estimates so there is no need to go through your caseload in detail.

This survey will take less than 5 minutes to complete. Your responses will be completely anonymous.

1. In all the patients you have seen, roughly what proportion have been referred **SPECIFICALLY** for COVID-related difficulties (e.g. worried about catching the virus or about postponed exams)?
   1. None
   2. Some
   3. About half
   4. Most all
2. Overall, how do you think COVID-19 has impacted your patients in the following domains?
   1. School
      1. More negative
      2. Both negative and positive
      3. More positive
      4. Don’t know
   2. Family life
      1. More negative
      2. Both negative and positive
      3. More positive
      4. Don’t know
   3. Friendship
      1. More negative
      2. Both negative and positive
      3. More positive
      4. Don’t know
   4. Treatment of physical illness
      1. More negative
      2. Both negative and positive
      3. More positive
      4. Don’t know
   5. Their own mental health
      1. More negative
      2. Both negative and positive
      3. More positive
      4. Don’t know
   6. Their parent’s mental health
      1. More negative
      2. Both negative and positive
      3. More positive
      4. Don’t know
3. Overall, what do you think was the main impact of COVID-19 on your patients?

E.g., not being able to see your face due to PPE, worrying about catching the virus, school closure, postponed school exams, delayed surgery, delayed psychological assessment/treatment, no longer being able to have visitors in the hospital *(open question)*

1. Although we are aware it varies for different patients, for those patients with **SPECIFIC**concerns related to COVID-19 or its impacts (e.g., catching the virus, missing exams), on average what proportion of session time is spent on those concerns? (*Slider 0-100)*

0 would indicate none of the session time

50 would indicate half of the session time

1. uld indicate all of the session time
2. Have you been providing low-intensity interventions SPECIFICALLY for COVID-related difficulties? i.e. One-off supportive telephone calls or a few (less than 6) telephone calls of 20-30 mins or less
   1. Yes
   2. No
3. If you selected 'Yes', what low-intensity interventions have you been providing? Please select all that apply.
   1. One-off supportive telephone calls
   2. A few (less than 6) telephones call of 20-30 minutes or less
   3. Signposting
4. If you selected multiple types of support, which was the most common support provided?
   1. One-off supportive telephone calls
   2. A few (less than 6) telephones call of 20-30 minutes or less
   3. Signposting
   4. NA
5. If you selected 'No', please explain why? *(Open question)*
6. How many patients have you provided low-intensity interventions to so far SPECIFICALLY for COVID-related difficulties? (*Open question)*
7. Where were these patients referred from? Please select all that apply.
   1. Within your own team
   2. Another psychological service teams
   3. Medical specialist/consultant
8. What problem(s) are you supporting patients for with low-intensity interventions? Please select all that apply
   1. Behavioural difficulties
   2. Emotional difficulties
   3. Other
9. If you selected ‘Other’, please explain. *(open question)*
10. Were standardised measures administered to these patients? E.g. RCADS
    1. Yes
    2. No
11. If you selected 'Yes', please state which measure(s) were completed. *(open question)*
12. How much support do you feel you need with providing low-intensity interventions to patients and their families SPECIFICALLY for COVID-related difficulties?
    1. No support needed
    2. Some support needed
    3. A lot of support needed
13. Is there anything else you would like to tell us about the psychological or emotional support you have been providing to patients and their families during COVID-19? *(open question)*
14. Lastly, what medical stream are you from? *(open question)*

Thank you for your input!

If you have any questions, please feel free to drop us a message at MentalHealth.Access@gosh.nhs.uk

**Appendix S3.**

*Table summarising clinical psychologists and assistant psychologists’ perspectives on the main impact of COVID-19 on patients and families*

| Theme | Frequency | Description | Quotes |
| --- | --- | --- | --- |
| **Theme: Social isolation** |  | This theme describes the specific impacts of social isolation on patients and families. |  |
| Sub-theme: Disconnect from the outside world | 20 | Many respondents described social isolation and its impact on various domains of patients’ and families’ lives, including patients being unable to interact with friends face-to-face, parents unable to see their children who are in foster care, and significantly reduced time outside the home due to social distancing. Respondents described patients and families feeling disconnected from the outside world as a result. | *“Not having face-to-face contact with parents and siblings – all my clients are in foster care.”*  *“I worry about them feeling connected to the outside world though.”* |
| Sub-theme: Loss of routine | 11 | Respondents reported patients expressing a loss of routine due to rapid changes in daily life in response to COVID-19. Patients have had to change and adapt their routine, and been unable to engage in valued and meaningful activities as per usual. Some patients with found this loss of routine difficult, and other patients experienced boredom. | *“Not having normal routine, such as schools, clubs.”*  *“Not being able to participate in sports and fun activities (e.g. football, swimming).”* |
| **Theme: School closure** |  | This theme highlights the specific impact of school closure on patients and families. |  |
| Sub-theme: Access to education | 22 | Respondents detailed how school closure has been a barrier to many patients’ access to education, such as lack of learning support at home for patients with learning difficulties, issues with remote school learning, parents finding home schooling hard, and difficulties with liaising with schools. However, school closure was not reported to be a barrier for all patients and families. One respondent reported that doing schoolwork at home was a very positive experience for some families. | *“School closure – both parents and children reported that home schooling was a challenge particularly when the child had educational needs as a result of their illness.”*  *“Many have found doing schoolwork in this environment very positive.”* |
| Sub-theme: Reduced school-specific stressors | 13 | Respondents said many patients experienced reduced stress, as a direct result from school closures. Many patients experience bullying from peers and receive questions about their physical illnesses, as well as mobility issues and worries about school performance. School closure has eliminated these school-specific stressors and reduced patients’ anxiety around school. | *“Many have visible differences so have been delighted to be at home and not having to deal with curiosity/questions and bullying.”* |
| Sub-theme: Exams | 10 | Respondents reported mixed responses to exams being cancelled due to COVID-19. Many reported patients worrying about exam results and its uncertainty, whereas others described patients feeling relieved about not having to sit them this year. | *“I had 1 teenager who was very worried that she would not be given the results she hoped for, but. She was very relieved not to have to sit exams.”* |
| Sub-theme: Anticipatory anxiety of returning to school | 11 | Respondents described anticipatory anxiety of returning to school in a lot of patients. This was especially the case for those who did not enjoy school and developed new routines at home over a long period of time. Families are also concerned about the uncertainty of how transitioning back to school will be like. | *“Other parents reported that children who had not enjoyed school prior to COVID-19 had seen a reduce in stress during lockdown, however raised concerns about coping when returning to school.”* |
| **Theme: Family relationships** |  | This theme describes the impact of COVID-19 on family relationships in patients and families. |  |
| Sub-theme: Pressure on the household | 11 | Respondents wrote about increased stress and pressure on family relationships. This was a result of spending a lot of time together with limited opportunity for private space. Moreover, pressures on relationships were more apparent in families with poor home environments before COVID-19, exacerbating embedded home issues. | *“Although some have thrived from being at home, this has understandably put additional pressure and strains on family relationships, and I have known some family relationships breakdown due to spending so much time together.”*  *“All family members being at home, some of which live in unsuitable home environments and this impacted on everyone’s stress and wellbeing.”* |
| Sub-theme: Improved relationships | 4 | Respondents reported that some families saw improved relationships during this time. Spending more time together promoted bonding and engaging with activities that families would normally not have the time to do. | *“Other parents talked to me about really enjoying having the whole family together and being able to do things they never normally get time to do.”* |
| **Theme: Physical health** |  | This theme describes the impact of COVID-19 on patients’ physical health. |  |
| Sub-theme: Improvements in physical health | 3 | Respondents described how patients have seen improvements in their physical health conditions due to spending more time on its management. | *“For a lot of the patients I’ve seen, it’s actually improved their physical condition due to more time and attention being taken with the management of their physical condition.”* |
| Sub-theme: Worsening of physical health | 3 | Respondents stated some patients experiencing worsening of their physical health conditions due to reduced activity levels during lockdown. | *“Reduced activity levels due to lockdown (and subsequent increase in pain presentations).”* |
| Sub-theme: COVID-19 hospital admissions | 2 | Respondents wrote about some patients who have contracted COVID-19 and had to deal with the associated difficulties like inflammation and hospital admissions. | *“As a team, we have been providing support to the patients with paediatric inflammatory multisystem syndrome (PIMS).”* |
| **Theme: Mental health** |  | This theme describes the impact of COVID-19 on patients and families’ mental health. |  |
| Sub-theme: Anxiety | 28 | Respondents wrote that many patients and families experienced increased anxiety during this time. Anxiety revolved different topics, including catching COVID-19, spreading the virus to vulnerable family members, leaving the house especially after shielding for a period of time, and wearing masks. The media was described by one respondent as a partial contributor to the anxiety as it caused confusion and concern in families. Another respondent also described families having had traumatic experiences from contracting the virus and being hospitalised in addition to their physical health deterioration. | *“Anxiety about reducing social distancing measures by coming out of shielding.”*  *“Some patients are in a high risk category and worried about catching the virus.”*  *“Some worry about catching the virus and passing it onto vulnerable family members.”*  *“Health anxiety after having COVID-19 and requiring PICU admission.”* |
| Sub-theme: 1 carer policy | 13 | Respondents reported specific distress in patients and families from the one carer policy in the hospital which was put in place to restrict hospital footfall to control the virus outbreak within the hospital. This policy resulted in patients feeling more isolated whilst inpatient, being unable to be visited by siblings, only one parent/carer being able to visit patients, parents feeling less supported in patient care, parents worrying about reduced opportunity for attachment building with patient, and no other visitors. This was stressful and even traumatic for some families, as some families were unable to spend time with patients reaching end-of-life. | *“One carer rule in hospital (families are much less supported by their own support networks, struggling more, isolated for young people and families.”*  *“Parents struggling with the one carer rule – how both parents able to develop attachment to their newborn baby when only one is allowed contact?”* |
| Sub-theme: Increased parental burdens | 4 | Respondents described families feeling overwhelmed by their increasing demands and multiple roles during this time as parents. | *“When working with parents, the impact of managing multiple roles and associated demands.”* |
| Sub-theme: Feelings of being different | 2 | Respondents reported patients feeling further ostracised by needing to shield as a young person because of their physical health conditions. This exacerbated feelings of being different. | *“More feelings of difference as a shielding young person.”* |
| Sub-theme: Low mood | 1 | One respondent described patients experiencing low mood. | *“Low mood…”* |
| Sub-theme: Behavioural difficulties | 1 | One respondent stated patients experiencing increased behavioural difficulties due to anxiety and stress. | *“Managing behavioural problems in children when there is increased anxiety/tension in the house due to having no breaks from each other or stress related to COVID-19.”* |
| Sub-theme: Improvements in mental health | 5 | Respondents reported some patients seeing improvements in their mental health and wellbeing due to reduced stressors in staying at home. | *“Some patients have reported benefits to their wellbeing, e.g. no longer being worried about exams, reduced panic attacks as staying at home.”* |
| **Theme: Treatments** |  | This theme describes the impact of COVID-19 on treatment for patients in the hospital. |  |
| Sub-theme: Reduced quality | 4 | Respondents reported reduced quality of services in the hospital for patients and families, including not being able to see clinicians’ faces due to PPE, uncertainty around diagnosis, not understanding the prognosis of diagnoses, and reduced opportunity for patient-centred care, such as availability of specific foods for patients with restrictive diets. This was all noted to be barriers to the care patients and families receive. | *“Barrier of PPE. The uncertainty around the diagnosis and prognosis. Lots of follow-up and contact, in context of not understanding prognosis.”*  *“Availability of preferred foods was a major concern for families with children with restrictive and limited diet at outset of COVID.”* |
| Sub-theme: Medical treatments | 10 | Respondents described negative impacts on medical treatments in the hospital. Families had worries about face-to-face appointments and had difficulties with attending check-ups and appointments. There was a lot of uncertainty around surgeries as some respondents reported delays in scheduled treatments. This delay also extended to an overall delay in medical team services. | *“Not having dates for surgery has been a key issue, particularly for parents of babies awaiting primary lip/palate repair. Also our osteotomy patients who have been preparing for surgery for up to two-years are struggling with the not knowing.”* |
| Sub-theme: Psychological treatments | 13 | Respondents described negative impacts on psychological treatments in the hospital. Patients and families struggled with being unable to attend face-to-face appointments, and delayed neuropsychological and cognitive assessments. Respondents said they have had to reduce the number of one-to-one psychology sessions with patients and families. Many respondents reported issues with delivering treatment remotely, such as technical difficulties and limitations of doing exposure and behavioural experiments virtually. One respondents also stated difficulties with families accessing support from local CAMHS via referrals. | *“Delays to psychological assessment, e.g. cognitive assessments.”*  *“Not being able to carry out in vivo behavioural experiments (especially relating to social anxiety).”*  *“Difficulty accessing psychological support via referral to local CAMHS.”* |
| **Theme: Social support** |  | This theme describes the impact of COVID-19 on social support for patients and families. |  |
| Sub-theme: Reduced access to established social support systems | 7 | Respondents reported families experiencing reduced access to established social support systems within the family and the wider societal context; from family support to governmental policies. | *“Reduced participation and access (socially and educationally).”*  *“Feeling marginalised by society and government policies about social distancing.”* |
| Sub-theme: Reduced access to social/community services | 2 | Respondents described some patients and families experiencing reduced access to support from charities and social services. | *“All of the above, as well as not being able to access charity/third sector support.”* |
| Sub-theme: Cultural stigma | 1 | One respondent wrote about cultural-specific stigma around contracting COVID-19 that impeded on families’ access to social support. | *“Some cultural specific concerns around stigma of having had COVID-19 preventing talking with support network.”* |

**Appendix S4.**

*Table summarising clinical psychologists and assistant psychologists’ experiences of providing support to patients and families during COVID-19*

| Theme | Frequency | Description | Quotes |
| --- | --- | --- | --- |
| **Theme: Remote/virtual support** |  | This theme highlights respondents’ experiences of delivering support for COVID-related difficulties through remote/virtual means, such as telephone or video sessions. |  |
| Sub-theme: Expectations | 2 | Generally, respondents reported how experiences of delivering support through remote/virtual sessions were positive and better than anticipated. These sessions were described to be successful and helpful for most patients, except for children with sensory impairments.” | *“Video therapy has gone far better than I had anticipated.”* |
| Sub-theme: Engagement | 3 | Respondents reported varied experiences of patient engagement in remote/virtual appointments. One described how the new format required creativity from respondents to ensure young people were engaged. Another respondent described engagement as a challenge. However, 1 respondent reported that remote/virtual sessions had no impact on patients’ engagement. | *“It has required creativity to engage some of the younger patients.”*  *“I even took on a brand new intervention case and it was all done on video. I was concerned that it would affect engagement, but it was fine.”* |
| Sub-theme: Access to psychological support | 5 | Respondents described how many young people have responded well to the remote/virtual format, and increased access to psychological support for patients and families. Reasons include patients not having to travel to the hospital and feeling safer at home. Simultaneously, the remote/virtual format has reduced access to psychological support for many patients with sensory impairments. | *“Some families who live far away have welcomed remote sessions as it means they can access support without making a long journey.”*  *“For families with children with hearing impairment or voice problems, using technology (phone or video sessions) has been difficult – to the point where some input has been put on hold until I can see them face-to-face again. The move to more remote working has the potential to disadvantage these youngsters.”* |
| Sub-theme: Technological challenges | 4 | Respondents’ experiences of using technology was mixed. One respondent stated that they have never delivered care through video conference calls before. One respondent found it easy to provide support using technology, while another found that remote/virtual sessions introduced added difficulty. | *“It has been extremely easy to provide the support due to [electronic system] and Zoom technology.”*  *“Support has needed to be offered primarily via Zoom or telephone. This comes with added complications and challenges.”* |
| Sub-theme: Distrust in remote/virtual working | 1 | One respondent described families being dissatisfied with the remote/virtual format and feeling as if the quality of support was reduced as a result. | *“Families finding it difficult to accept the findings of the assessments because they do not feel that the remote assessment has been thorough enough.”* |
| **Theme: Workload** |  | This theme highlights changes in respondents’ workloads in response to COVID-related difficulties and arising needs. |  |
| Sub-theme: Increased workload | 8 | Respondents described an influx in their workload due to new cases being referred for COVID-related difficulties. However, current patients also required additional support for COVID-related difficulties, unrelated to the difficulties that respondents were treating them for initially. This resulted in respondents providing additional sessions. Some described changes in workload and mental health difficulties that were parallel to changes in governmental policies and regulations. | *“Huge increase in our workload.”*  *“Though the numbers requiring psychological support have vastly increased the workload, it is a temporary blip as lockdown restrictions are easing.”* |
| Sub-theme: Adapting to patient needs around COVID-19 | 3 | Respondents reported having to adapt to and support new presenting issues as a result of COVID-19, as well as managing the implications of the impact of COVID-19 in patients and families and supporting their coping. | *“For some families it was the parents' anxiety about COVID-19 that was evident and intervention was about normalising this anxiety, trying to contain the uncertainty, and once lockdown ended (or rather shielding finished) helping them prepare for going outside again.”* |
| **Theme: Facilitators and barriers** |  | This theme describes the personal and organisational factors that act as facilitators and barriers to delivering support for COVID-related difficulties to patients and families. |  |
| Sub-theme: Teamwork and communication | 5 | Respondents described the importance of working together and communicating effectively with colleagues to support patients and families. This included liaison within their own teams and across different teams and services within the hospital. One respondent also reported working closely with local services outside of the hospital. | *“Teams have really pulled together so communication has been far better over the past few months, which all goes towards supporting families.”*  *“We’ve been doing a bit more liaison for vulnerable families with their local teams to problem solve issues which come up.”* |
| Sub-theme: Motivation | 1 | Despite the increased workload and pressures for teams and services, 1 respondent described wanting to provide support to patients. | *“Providing support to the patients… obviously we wanted to provide it.”* |
| Sub-theme: Electronic system | 1 | One respondent reported that the online electronic system that patients and families can access was helpful for administrative tasks for appointments. | *“[Electronic system] has been a great tool in getting questionnaires sent to families.”* |
| Sub-theme: Lack of face-to-face care | 1 | One respondent described the lack of face-to-face appointments as an impediment in the understanding and intervention of patients’ difficulties. | *“Lack of availability of meeting patients for meeting face to face for assessments and treatments, which limited our understanding and ability to intervene with some patients.”* |
| Sub-theme: Reduced input from other services | 1 | One respondent stated that due to the reduced input from other services as a result of COVID-19, the work they do with patients and families in the hospital was affected. | *“Children on child protection plans are having fewer visits from social workers and it is harder to monitor their wellbeing.”* |
